# Isolation and characterization of *Ehrlichia chaffeensis *RNA polymerase and its use in evaluating p28 outer membrane protein gene promoters

**DOI:** 10.1186/1471-2180-11-83

**Published:** 2011-04-22

**Authors:** Bonto Faburay, Huitao Liu, Lalitha Peddireddi, Roman R Ganta

**Affiliations:** 1 Department of Diagnostic Medicine/Pathobiology, College of Veterinary Medicine, Kansas State University, Manhattan, KS 66506, USA

## Abstract

**Background:**

*Ehrlichia chaffeensis *is a tick-transmitted rickettsial pathogen responsible for an important emerging disease, human monocytic ehrlichiosis. To date how *E. chaffeen*sis and many related tick-borne rickettsial pathogens adapt and persist in vertebrate and tick hosts remain largely unknown. In recent studies, we demonstrated significant host-specific differences in protein expression in *E. chaffeensis *originating from its tick and vertebrate host cells. The adaptive response of the pathogen to different host environments entails switch of gene expression regulated at the level of transcription, possibly by altering RNA polymerase activity.

**Results:**

In an effort to understand the molecular basis of pathogen gene expression differences, we isolated native *E. chaffeensis *RNA polymerase using a heparin-agarose purification method and developed an *in vitro *transcription system to map promoter regions of two differentially expressed genes of the p28 outer membrane protein locus, *p28-Omp14 *and *p28-Omp19*. We also prepared a recombinant protein of *E. chaffeensis *σ70 homologue and used it for *in vitro *promoter analysis studies. The possible role of one or more proteins presents in *E. chaffeensis *lysates in binding to the promoter segments and on the modulation of *in vitro *transcription was also assessed.

**Conclusions:**

Our experiments demonstrated that both the native and recombinant proteins are functional and have similar enzyme properties in driving the transcription from *E. chaffeensis *promoters. This is the first report of the functional characterization of *E. chaffeensis *RNA polymerase and *in vitro *mapping of the pathogen promoters using the enzyme. This study marks the beginning to broadly characterize the mechanisms controlling the transcription by *Anaplasmataceae *pathogens.

## Background

*Ehrlichia chaffeensis *is an obligate intracellular rickettsial pathogen and the causative agent of an important emerging zoonotic disease, human monocytic ehrlichiosis [1-4]. This *Amblyomma americanum *tick-transmitted pathogen causes infections in susceptible hosts (humans), host reservoirs (white-tailed deer), and less well described hosts such as the dog, goat and coyote [5-10]. *E. chaffeensis *has an unusual developmental cycle that requires growth and replication within eukaryotic cells of vertebrate and tick hosts [11]. During its developmental cycle, there is conversion between two distinct morphological forms, the elementary bodies (EBs) and reticulate bodies (RBs) [12,13]. The EBs are the infectious form and upon entry into a host cell, they differentiate into metabolically active reticulate bodies (RBs), which are larger compared to EBs and divide by binary fission [12-14]. The reticulate bodies are also non-infectious forms [14]. Later in the developmental cycle, RBs convert back to EBs, which are released from infected cells [12,14]. The transformation of RBs to EBs by *E. chaffeensis *is observed in both vertebrate and tick hosts [15].

The mechanism by which the pathogen survives in dual hosts by adapting to changes in different host environments is unclear. Recent studies described the differential gene and protein expression profiles of the pathogen originating from tick and mammalian cell environments [15-18]. Moreover, *E. chaffeensis *organisms recovered from infected tick cells produce longer-lasting infections in mice compared to the infection with organisms harvested from mammalian macrophages [19]. Differentially expressed proteins of *E. chaffeensis *included the predominant expression from outer membrane protein genes *p28-Omp19 *and *p28-Omp14 *in mammalian and tick cell environments, respectively [15-19]. The adaptive response to different host environments requires altering the gene expression, often regulated at the transcriptional level by altering RNA polymerase (RNAP) activity [20]. A typical bacterial RNAP consists of five polypeptide chains; two α subunits, one each of β and β' subunits, and a σ subunit. The enzyme can take two forms, a holoenzyme containing all four different subunits or core polymerase that lacks a σ subunit [21]. The capacity to synthesize RNA resides in the core polymerase and the role of a σ subunit is to direct initiation of transcription from specific promoters [22,23]. The genome of *E. chaffeensis *includes two sigma factor genes; the homologs of the major bacterial sigma factor, σ^70^, and an alternative sigma factor, σ^32 ^[24]. The current lack of established methods to stably transform, transfect, conjugate, or electroporate *E. chaffeensis *remain a major limiting factor to study mechanisms of gene expression by traditional methods. Mapping the functions of *E. chaffeensis *genes *in vivo *cannot be performed because genetic manipulation systems are yet to be established. To overcome this limitation, in a recent study we reported the utility of *Escherichia coli *RNAP as a surrogate enzyme to characterize *E. chaffeensis *gene promoters [25]. Although the *E. coli *RNAP proved valuable for mapping *E. chaffeensis *gene promoters, the extrapolation of the data requires further validation using the *E. chaffeensis *RNAP.

In this study, we developed a functional *in vitro *transcription system by utilizing G-less transcription templates [26] to drive transcription from two *E. chaffeensis *promoters. We described the partial purification and characterization of *E. chaffeensis *RNAP and its use in characterizing the transcriptional profiles of two p28-Omp gene (*p28-Omp*) promoters. In this study, we also described the recombinantly expressed *E. chaffeensis *sigma factor, σ^70^, and its use in promoter analysis studies after its reconstitution with *E. coli *core enzyme. Modulatory effect of *E. chaffeensis *protein lysates on *in vitro *transcription is also described in this study to serve as the first step towards determining the regulatory mechanisms underlying gene expression in this pathogen.

## Results

### Isolation of *E. chaffeensis *RNA polymerase (*E. chaffeensis *RNAP)

**Figure 1:**
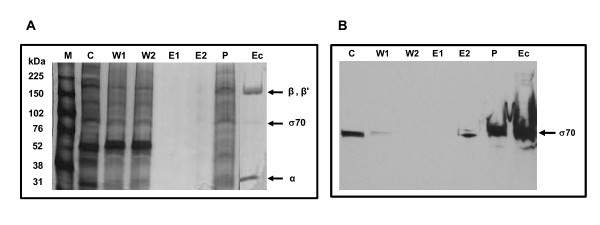
***E. chaffeensis *RNA polymerase purification by employing heparin agarose column purification method**. A) Silver-stained SDS-PAGE gel profile of heparin agarose purified fractions of *E. chaffeensis *RNA polymerase. M, protein standards (kDa); C, *E. chaffeensis *crude lysate; W1, first wash fraction from the column; W2, second column wash; E1, first elution fraction; E2, second elution fraction; P, pooled dialyzed fractions of eluted fractions 3 to 6; Ec, *E. coli *holoenzyme from Epicenter^® ^B) Western blot analysis of the proteins resolved in panel A with *E. coli *anti-sigma70 monoclonal antibody, 2G10.

**Figure 2:**
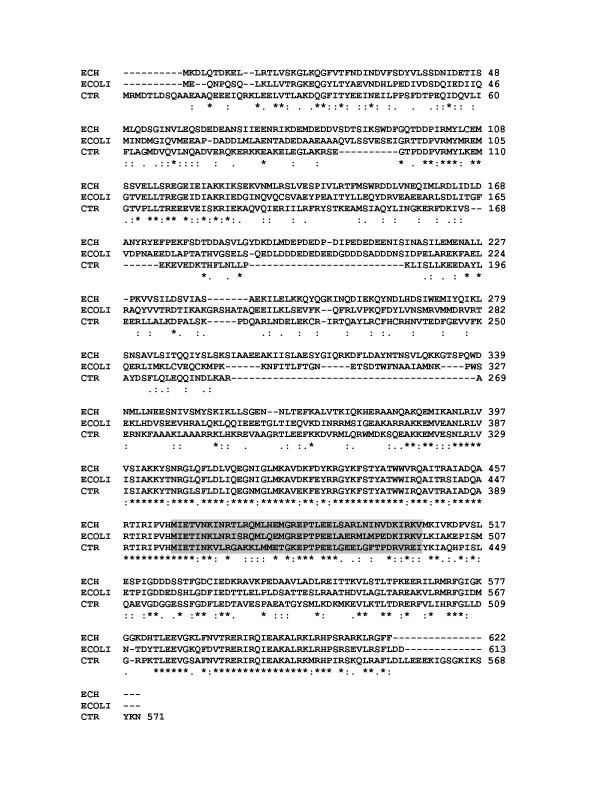
**Comparative alignment of complete amino acid sequences of *E. chaffeensis *(ECH), *E. coli *(ECOLI) and *C. trachomatis *(CTR) major σ subunit to show sequence homology**. The GenBank accession numbers for these sequences are NC007799, NC000913 and NC012687, respectively. The numbers of the amino acids of the corresponding genus are indicated at the far right. Asterisks denote amino acid homology; dots denote amino acid mismatch. Dashes are gaps introduced into the sequence to improve the alignment. The shaded amino acid sequence represents the putative binding site of the *E. coli *anti-σ^70 ^monoclonal antibody, 2G10 [29].

*E. chaffeensis *DNA-dependent RNA polymerase (*E. chaffeensis *RNAP) was partially purified from the organisms grown in macrophage cultures by adapting heparin-agarose column purification method described earlier for other bacterial systems [27]. To determine the purity and polypeptide composition of the *E. chaffeensis *RNAP, several eluted fractions were electrophoresed on a polyacrylamide gel that was stained using silver nitrate (Figure 1A). The gel pattern revealed that the *E. chaffeensis *RNAP had a subunit structure similar to *E. coli *RNAP (that is also typical of other eubacteria) with five major subunits (α_2_, β, β', σ). Western blot analysis confirmed the presence of *E. chaffeensis *σ^70 ^polypeptide when assessed using a heterologous *E. coli *anti-σ^70 ^monoclonal antibody, 2G10 (Figure 1B). Amino acid alignment of the sequence of *E. chaffeensis *σ^70 ^polypeptide with *E. coli *σ^70 ^polypeptide revealed significant homology which also spanned to the putative binding site sequence of 2G10 antibody to *E. coli *σ^70 ^polypeptide [28,29] (Figure 2). The homology between amino acid residues of σ^70 ^polypeptides recognised by 2G10 antibody [28] is considerably higher between *E. chaffeensis *and *E. coli *than between *E. chaffeensis *and *Chlamydia trachomatis *. Protein BLAST search (at National Center for Biotechnology Information Bethesda, MD, USA) of the putative amino acid binding site sequence of 2G10 in *E. coli *[28,29] against *E. chaffeensis *(Arkansas isolate) genome identified only one significant match (E-value of 1e^-11 ^and having 69% identity) with *E. chaffeensis *RNAP σ^70 ^polypeptide, RpoD.

**Figure 3:**
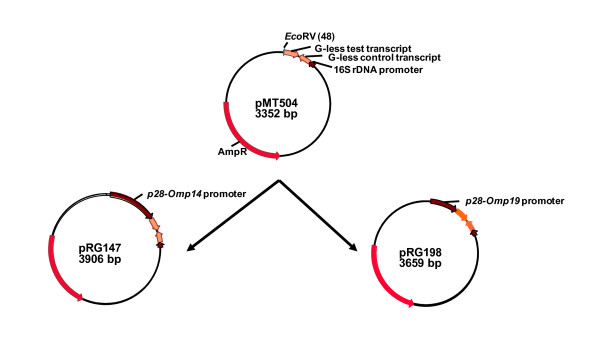
**Construction of transcription plasmids, pRG147and pRG198**. The plasmids were constructed by cloning PCR-amplified *E. chaffeensis*-specific promoters of *p28-Omp14 *(pRG147) and *p28-Omp19 *(pRG198) into the EcoRV located upstream of a G-less cassette in pMT504 [26].

**Figure 4:**
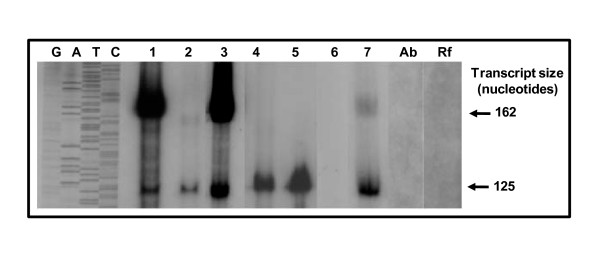
**Transcriptional analysis of *E. coli *and *E. chaffeensis *RNAPs using *p28-Omp 14 *and *19 *promoters**. Transcription of pRG147 (lane 1), pRG147R (lane 2), pRG198 (lane 3), pRG198R (lane 4), pMT504 (lane 5) was assessed using *E. coli *holoenzyme or with *E. coli *core enzyme with pRG198 (lane 6). Transcription of pRG198 by HA-purified *E. chaffeensis *RNAP (lane 7); inhibition of transcription of *E. chaffeensis *RNAP by the addition of 4 μg of 2G10 (Ab); inhibition of transcription of *E. chaffeensis *RNAP by addition of 25 μg/ml of rifampin (Rf). Inhibition assays were performed using pRG198. GATC, sequencing ladder generated using the plasmid pRG198 sequenced with primer M13F-40 to serve as the molecular weight markers.

**Figure 5:**
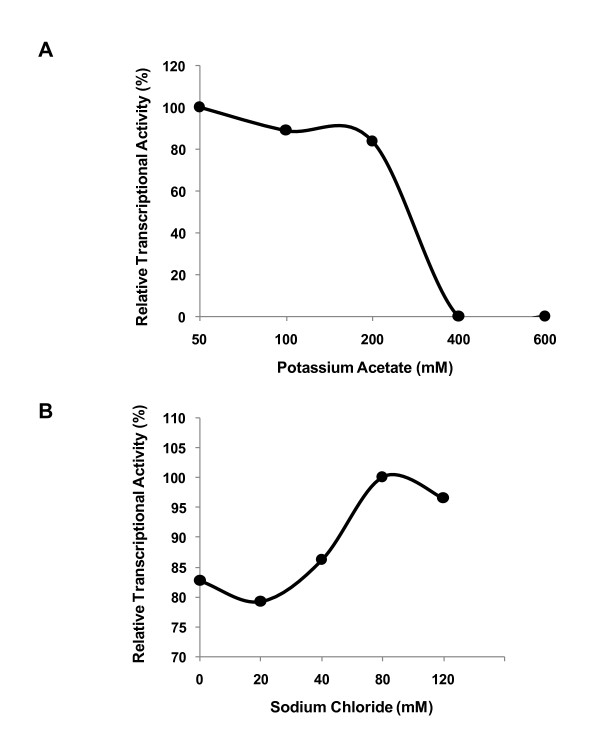
***In vitro *transcription showing the effect of varying salt concentrations of potassium acetate (Panel A), and sodium chloride (panel B)**. Transcription of the *p28-Omp19 *promoter region in pRG198 plasmid was assessed using the purified *E. chaffeensis *RNAP.

In support of testing the functionality of *p28-Omp14 *and *p28-Omp19 *gene promoters, we constructed *in vitro *transcription templates, pRG147 and pRG198, by cloning the promoter regions of the genes into the pMT504 plasmid (Figure 3). The plasmid pMT504 is a G-less cassette containing two transcription templates cloned in opposite directions to aid in driving transcription from promoters introduced upstream of the G-less cassette sequences [26]. (The promoter segments were amplified from *E. chaffeensis *genomic DNA using the primers listed in Table 1.) The functionality of the promoters of *p28-Omp14 *and *p28-Omp19 *in correct orientation, in plasmids pRG147 and pRG198, was initially confirmed using *E. coli *holoenzyme containing its σ^70 ^polypeptide (Figure 4). Subsequently, transcriptional activity of the heparin-agarose purified RNAP fractions was evaluated. *E. chaffeensis *RNAP activity was detected in purified pooled fractions (data shown for pRG198 in Figure 4). The purified enzyme is completely inhibited in the presence of anti-σ^70 ^monoclonal antibody, 2G10, or in the presence of rifampicin (Figure 4). Further characterization using varying salt concentrations showed that the enzyme was active in presence of potassium acetate up to 200 mM concentration and was inhibited at 400 mM (Figure 5A), and the optimum concentration for activity of the enzyme for sodium chloride was observed at 80 mM (Figure 5B).

**Table 1:** Primer pairs and probes used in this study

Primers/probes	Sequence	^**1**^**Orientation**	Reference
**For cloning *p28-Omp14 and p28-Omp19 *promoters into pMT504**			

RRG217 (p28-Omp14)	5"-ttgctcaaccataaaataatggga	F	25

RRG695 (p28-Omp14)	5"-taaaaatttaagaataatgaaag	R	This study

RRG185 (p28-Omp19)*^#^	5"-GACTCTAGA***cttttaa***ttttattattgccacatg	F	25

RRG696 (p28-Omp19)	5"-aaataaattaacaatagtagaag	R	This study

**For cloning RpoD gene into pET32**			

RRG742*	5"-*GAGCCATGG*cttaacaaattctatattttccctaactc	F	This study

RRG743*	5"-*CGCTCGAG*ttaactattgatattacaatgacctagt	R	This study

**For TaqMan RT-PCR of test G-less transcripts**			

RRG766	5"-ccttcctccatctataccac	F	This study

RRG767	5"-gagagtgaatgatgatagatttg	R	This study

RRG765 (TaqMan Probe)	5"-cattattcctcctatcttctcctcttctc		This study

**For TaqMan RT-PCR of control G-less transcripts**			

RRG769	5"-tactcacccaatactcccta	F	This study

RRG770	5"-gtggaatgagaaatgagtgt	R	This study

RRG768 (TaqMan Probe)	5"-cttatcctctcctcacctctccctc		This study

**For sequencing pRG198**			

M13F-40	5"-gttttcccagtcacgac		Commercial

***p28-Omp14 *promoter EMSA probes**			

**Full length probe**			

RRG 217**		F	

RRG 218	5' gttaataaaccttttataaaag	R	25

**Probe 1 (P1)**			

RRG217**		F	

RRG623	5"-ggtttagccattttaaatgtg	R	This study

**Probe 2 (P2)**			

RRG267	5"-cagttaactttctgtaaacttc	F	25

RRG623	5"-ggtttagccattttaaatgtg	R	This study

**Probe 3 (P3)**			

RRG269	5"-cgttttctgctttattagaatg	F	25

RRG625	5"-gtacatgcattatgagcaaatc	R	This study

**Probe 4 (P4)**			

RRG270	5"-gttccgtatttattaatatatg	F	25

RRG626	5"-ctatacttaactttactactta	R	This study

**Probe 5 (P5)**			

RRG272	5"-ggataagtactttagcaagtgg	F	25

RRG627	5"-gtctagaatataaaatttctttc	R	This study

***p28-Omp19 *promoter EMSA probes**			

**Full length probe**			

RRG 185**		F	25

RRG 445	5' atataacctaatagtgacaaataaattaac	R	This study

**Probe 6 (P6)**			

RRG185**		F	25

RRG628	5"-gcacttataaactagtccc	R	This study

**Probe 7 (P7)**			

RRG276	5"-gtgctgtttttctcacctttacac	F	25

RRG629	5"-cttttgtaaggaaaatttaatata	R	This study

^1^F, forward primer; R, reverse primer

* Text in capital letters refers to sequences inserted for creating restriction enzyme sites

^#^Text in bold and italics letters refers to 7 nucleotides of coding sequence from the 3" end of p28-Omp18 gene used in the primer

** Primer sequences were presented only once when a primer was described for the first time.

### *In vitro *transcription by recombinant *E. chaffeensis-*σ^70^

**Figure 6:**
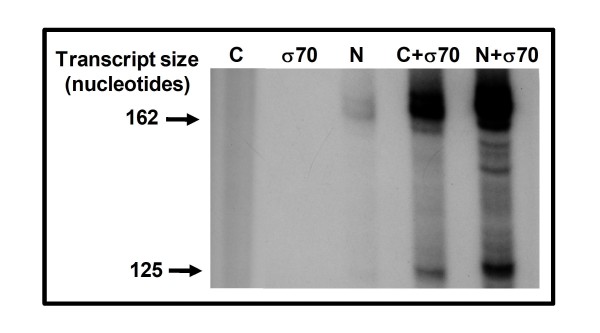
**Transcriptional analysis of recombinant *E. chaffeensis*-σ^70 ^using pRG198 transcriptional template**. C, transcription products by *E. coli *core enzyme alone; σ^70^, transcription products by the recombinant *E. chaffeensis *σ^70 ^protein; N, transcription products by purified *E. chaffeensis *RNAP; C + σ^70^, transcription products by by *E. coli *core enzyme saturated with recombinant *E. chaffeensis *σ^70^; N + σ^70^, transcription products by native purified enzyme saturated with recombinant *E. chaffeensis*-σ^70^.

**Figure 7:**
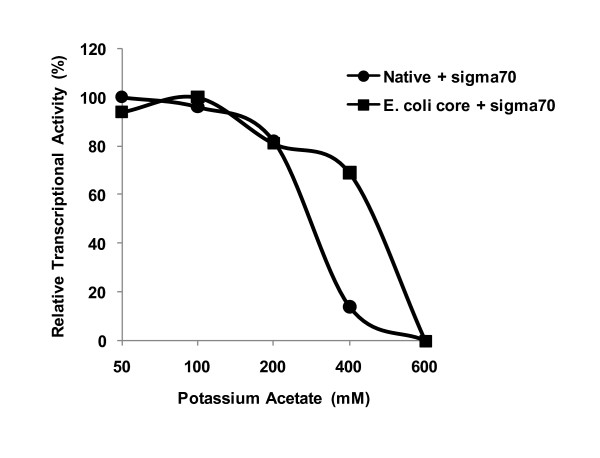
**Transcription of pRG198 with varying potassium acetate concentrations showing transcription by *E. chaffeensis *RNAP saturated with the recombinant σ^70 ^and by *E. coli *core RNAP reconstituted with recombinant σ^70^**.

We reconstituted *E. coli *RNAP core enzyme with recombinantly expressed *E. chaffeensis *σ^70 ^and the resulting holoenzyme effectively transcribed the promoters of *p28-Omp14 *and *p29-Omp19 *(data presented for *p28-Omp 19 *promoter in Figure 6), but the core enzyme alone or recombinant *E. chaffeensis *σ^70 ^alone did not drive the transcription. Saturation of the purified enzyme with recombinant σ^70 ^also resulted in enhanced transcriptional signals (Figure 6). General transcriptional profile of both the reconstituted enzymes in the presence of varying potassium acetate concentrations were similar (Figure 7), although a relatively stronger transcriptional signal at 400 mM salt concentration was detected for *E. coli *core enzyme saturated with *E. chaffeensis *recombinant σ^70 ^subunit (Figure 7).

### Modulation of *E. chaffeensis *RNAP activity by whole-cell protein

We evaluated the effect of *E. chaffeensis *whole-cell protein lysate, prepared from the bacteria grown in macrophage cell line, on transcription of *p28-Omp14 *and *p28-Omp19 *constructs using the native purified enzyme. The resulting transcripts were analyzed by two independent methods; densitometry of radiolabeled transcripts and the Taq-Man probe-based, real-time RT-PCR. These analyses showed enhanced transcriptional activity in the presence of 4 μg of *E. chaffeensis *whole-cell lysate. Densitometric analysis revealed a 1.8-fold increase in transcriptional signal for the *p28-Omp14 *promoter construct and a 2.1-fold increase for *p28-Omp19 *construct (Table 2). Addition of the same amount of protein yielded a similar fold increases when transcription was assessed with *E. coli *core enzyme saturated with *E. chaffeensis *recombinant σ^70^. No transcription occurred with the addition of whole-cell lysate alone in the absence of an enzyme, a potential source of *E. chaffeensis *RNAP. Similarly, the addition of boiled lysate did not cause any change in transcriptional signals. Quantitation by real-time RT-PCR for the calculation of fold increase in transcription in the presence of *E. chaffeensis *whole-cell protein lysate was carried out as described previously [30,31]. Transcription of *p28-Omp19 *construct with purified *E. chaffeensis *RNAP, as quantified by real-time RT-PCR, showed a 2.24 fold enhancement in the presence of 4 μg of the protein lysate, whereas transcription of *p28-Omp14 *promoter construct resulted in a 1.81 fold-enhancement (Table 2), indicating a higher degree of agreement between the data generated by densitometric and real-time RT-PCR methods of quantitation (Table 2).

**Table 2:** Effect of macrophage-culture grown *E. chaffeensis *whole-cell lysate on the transcription of *p28-Omp14 *(pRG147) and *p28-Omp19 *(pRG198) promoter constructs quantitated by densitometry and real-time RT-PCR (fold change)

	**Densitometry**^#^				Real-time RT-PCR			
**Template**	**no lysate** **(x ± std)**	**with lysate** **(x ± std)**	**fold increase**	*p*-value*	**no lysate** **(Ct ± std)**	**with lysate** **(Ct ± std)**	**fold increase**	*p*-value*

pRG147	83.3 ± 1.8	45.5 ± 1.9	1.8	<0.0001	17.51 ± 0.81	16.64 ± 0.23	1.81	0.0174
pRG198	76.9 ± 1.7	35.7 ± 1.6	2.2	<0.0001	17.48 ± 0.08	16.27 ± 0.06	2.24	0.0013

^#^volume of the unoccupied space available under the signal is quantitated

*p-value of ≤ 0.05 is significant

### EMSA analysis of upstream sequences of *p28-Omp14 *and *p28-Omp19 *promoters

**Figure 8:**
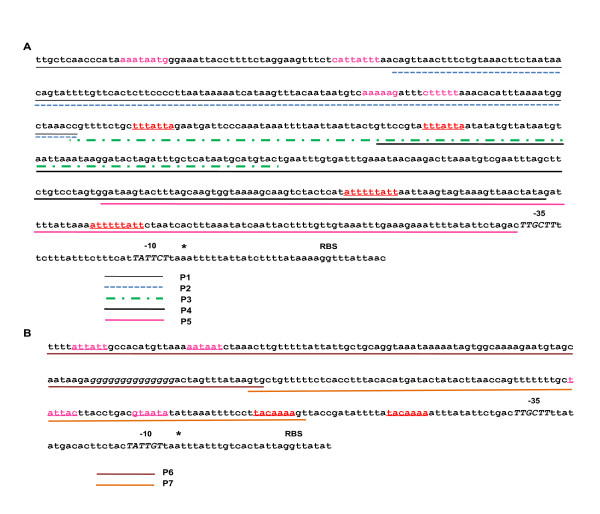
**Sequences of EMSA probes used in this study**. Sequences of *p28-Omp14 *P1-P5 (panel A) and *p28-Omp19 *P6 and P7 (panel B) represent promoter segments utilized in the EMSA experiments.

**Figure 9:**
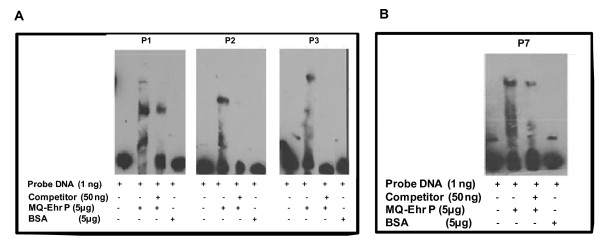
**EMSA using short segments of three biotin-labeled probes of *p28-Omp14 *(panel A) and one *p28-Omp19 *(panel B) promoter segments**. Addition of *E. chaffeensis *protein extracts (MQ-Ehr P) and unlabeled promoter DNA as a cold competitor (Competitor) or bovine serum albumin (BSA) as a non-specific protein control are indicated in captions at the bottom of the Figure for both the panels.

Electrophoretic mobility shift assay (EMSA) experiments utilizing the complete promoter regions of the *p28-Omp14 *and *p28-Omp19 *of *E. chaffeensis *showed promoter-specific binding of tick cell- or macrophage-derived *E. chaffeensis *proteins (not shown). Addition of 50 ng of specific competitor DNAs consisting of unlabeled full length promoter DNA of *p28-Omp14 *or *p28-Omp19 *abolished the shift of DNA-protein complex migration for both promoter regions. To further assess the interactions of *Ehrlichia *proteins with putative upstream sequences, five biotin-labelled short upstream DNA segments of *p28-Omp14 *(probes P1 to P5) (Figure 8A) and two DNA segments of *p28-Omp19 *(P6 and P7) (Figure 8B) promoters were prepared and used in the EMSA experiments. The promoter sequences of genes 14 and 19 included direct repeats and palindromic sequences [25]. The probes included one or more of the sequences. Three of the five probes for the *p28-Omp14 *promoter region exhibited significant shift in mobility in the presence of protein lysate from macrophage derived *E. chaffeensis *compared to the controls which contained probe alone with no lysate added or when non-specific protein was added to the probe fragments (Figure 9A). A shift in mobility was also noted in the interaction with one probe segment of the *p28-Omp19 *promoter region when the protein lysate was added (Figure 9B). Addition of a 50-fold excess of unlabeled specific-competitors in the binding reactions significantly reduced the mobility shift of the probes. Densitometry analysis of the mobility shifted fragments differed for each probe compared to the non-shifted fragments. The P1 probe had 84% shift which reduced to 29% when competitor DNA was added; P2 and P3 probes had about 31%, and 27% shifts, respectively, and the shifts for these probes were completely abolished in the presence of specific competitors. The *p28-Omp19 *promoter region probe had about 23% shift which was reduced to 10% in the presence of specific competitor.

## Discussion

A major impediment to the study of regulation of gene expression in the human monocytic ehrlichiosis pathogen, *E. chaffeensis*, is the absence of an experimental genetic manipulation system due to the inability to stably transform the organism. To partially overcome this constraint, we constructed plasmid transcription templates by transcriptional fusion of *p28-Omp14 *and *p28-Omp19 *promoters to a G-less transcriptional template and isolated *E. chaffeensis *RNAP to create a system for transcriptional analysis *in vitro*, similar to studies reported for *Chlamydia *species [20,26,32-35]. We adapted the bacterial RNAP purification methods reported in the literature [21,27,36,37] to recover functionally active *E. chaffeensis *RNAP. The procedure has been modified from a single-column purification method used for RNAP from *E. coli*, *Bacillus subtilis*, *Chlamydia trachomatis, Rickettsia prowazekii *and to recover the enzymes from several other bacterial organisms [21,27,37]. The purification steps involved the use of sodium deoxycholate, a bile salt often used in cell lysis but reportedly effective in the isolation of membrane proteins and in affinity chromatography by preventing non-specific binding [36]. This property may be critical for the recovery of active enzyme, since previous studies in *R. prowazekii*, a closely related species, showed that up to 62% of total RNAP activity was associated with membrane proteins [27]. The heparin-agarose purification step is known to remove RNAP inhibitors and endogenous DNA [27]. The recovered *E. chaffeensis *enzyme showed transcriptional activity for both *p28-Omp14 *and *p28-Omp19 *promoters and marked the first study describing RNAP activity of *E. chaffeensis*. SDS-PAGE profile suggested that the enzyme is partially pure and *E. chaffeensis *RNAP has a typical bacterial holoenzyme composition with five major subunits, α_2_, β, β', and σ.

The enhanced RNAP activity following addition of *E. chaffeensis *recombinant sigma 70 suggests that the preparation had less than stoichiometric amounts of the sigma factor, which is consistent with findings of the recovery of *E. coli *RNAP when employing similar procedures [22,27]. Previous studies suggest that RNAPs purified by heparin-agarose chromatography methods are only about 30% saturated with the major sigma factor, σ^70 ^[21] and do not co-purify with alternative sigma factors, such as a σ^32 ^homolog [20].

In this study, we presented evidence that the major *E. chaffeensis *sigma subunit, σ^70^, was also recognized by a heterologous *E. coli *anti-σ^70 ^monoclonal antibody, 2G10. Functional studies with the 2G10 suggest that this antibody can effectively inhibit *in vitro *transcriptional activity of *E. coli *[29] and *C. trachomatis *RNAP holoenzymes [28]. Similarly, this antibody inhibited the *E. chaffeensis *RNAP activity. These data are consistent with our bioinformatic analysis that the putative 2G10 antibody binding site sequences of *E. coli *and *E. chaffeensis *σ^70 ^subunits of RNAP share high degree of homology. Transcriptional inhibition of the enzyme by the anti- σ^70^monoclonal antibody and rifampin, a potent inhibitor of prokaryotic RNAP [27,38], demonstrates that the *in vitro *transcriptional activity in our study was due to the isolated *E. chaffeensis *RNAP.

Transcriptional profiles depicting salt tolerance of purified enzymes have been described for prokaryotes, such as, *C. trachomatis *and *E. coli *[20,39]. In *E. coli*, transcription of a σ^70^-regulated promoter decreases dramatically between 100 mM and 150 mM potassium acetate [39], whereas σ^66^-dependent promoter activity of *Chlamydia *is completely inhibited at 400 mM concentration [20]. The purified *E. chaffeensis *RNAP, reported in this study, also showed a similar range of salt tolerance as observed for other bacterial σ^70 ^dependent RNAPs. For example, the enzyme showed optimum transcriptional activity at 80 mM sodium chloride, a slight difference from the optimal 50 mM concentration reported for the *R. prowazekii *RNAP [27]. The minor differences in the salt tolerance properties may be unique to *E. chaffeensis *RNAP.

Previous studies suggest that RNAP fractions purified by heparin-agarose chromatography methods are typically about 30% saturated with the major sigma subunit [20]. Thus the presence of free core enzymes in the preparation allows reconstitution studies or saturation with recombinant sigma factors to enhance transcription *in vitro*. Thus we prepared a purified recombinant *E. chaffeensis *σ^70 ^subunit and used for assessing transcriptional activity by saturation of the native enzyme or by reconstitution with *E. coli *core enzyme. Saturation of the purified RNAP with the recombinant subunit resulted in enhanced transcriptional signals. Reconstitution of *E. coli *core enzyme with *E. chaffeensis *recombinant σ^70 ^subunit had similar salt sensitivities to that of purified *E. chaffeensis *RNAP before and after saturating with the recombinant subunit. These data are consistent with earlier reports indicating that purified *C. psittacci *σ^66 ^was effective in stimulating transcription by *C. trachomatis *and *C. psittaci *RNAP preparations [32] and highlights that *E. coli *core enzyme reconstituted with *E. chaffeensis *sigma factor offers an alternative approach to *in vitro *characterization of *E. chaffeensis *promoters as described for *C. trachomatis *[20,33]. Previously, we and others reported the use of *E. coli *system in characterizing the promoters of *E. chaffeensis *[25,40]. The current study offers an additional advantage over the *E. coli *system in that it uses *E. chaffeensis *RNAP or *E. coli *core enzyme with *E. chaffeensis *recombinant σ^70^.

Regulation of gene transcription in prokaryotes involves a complex network and is controlled at the stage of RNA synthesis in which transcription factors (TFs) are key components [41,42]. TFs play an important role in regulating the transcription of specific genes by acting on the DNA regulatory sequences within the gene promoters [41,42]. When a transcription factor binds to a specific promoter, it can either activate or repress transcription [35,43,44]. To investigate the possible modulatory role of *E. chaffeensis *proteins on transcription of promoters of two differentially expressed genes, *p28-Omp14 *and *p28-Omp19*, we prepared *E. chaffeensis *whole-cell protein lysate from macrophage-derived bacteria and evaluated its effect on transcription *in vitro*. Addition of the macrophage cell infection-derived *E. chaffeensis *protein extracts resulted in enhanced transcription suggesting that promoters of the *p28-Omp14 *and *p28-Omp19 *genes may be regulated in response to changing environments of the pathogen. Importantly, the enhanced *in vitro *transcription observed in this study in response to addition of protein extracts suggests that the lysates contain transcription regulators. Given the differential expression of *p28-Omp14 *and *p28-Omp19 *genes [15] in vertebrate and invertebrate hosts, the hypothesis that promoters of these genes may be under both positive and negative regulation in response to the changing host environments is also plausible. This hypothesis requires additional investigations, including the evaluation of the impact of tick cell environment. As an organism may express diverse array of transcription factors, it is highly likely that *E. chaffeensis *may regulate its gene expression via modulating the expression of transcription factors in support of maintaining its existence in dual hosts. Transcription regulation of a gene is a dynamic process and is responsive to environmental cues under which TFs trigger regulation [39,45-47]. This study shows the first evidence of stimulatory effect of *E. chaffeensis *whole-cell protein extract on the transcription of both *p28-Omp14 *and *p28-Omp19 *promoters *in vitro*. In our previous studies, we reported that the expression levels of the *p28-Omp14 *and *p28-Omp19 *genes are different in macrophage and tick cell environments [16,19]. Although both the genes are transcriptionally active in macrophage host cell environment under *in vitro *and *in vivo *conditions, the expression levels for *p28-Omp19 *is higher for the bacteria in infected macrophages, whereas in tick cells *p28-Omp14 *is the predominantly expressed protein [16,19]. Consistent with those observations, the promoter constructs of both *p28-Omp14 *and *p28-Omp19 *genes remained active and enhanced when *E. chaffeensis *protein lysates prepared from macrophage culture derived organisms were added. Additional investigations are needed to further define the differences in the expression levels for the *p28-Omp14 *and *p28-Omp19 *genes in macrophage and tick cell environments. A gene in a cell may be regulated by different TFs, and the contribution from different TFs may be variable under different environmental conditions [48]. Thus, more detailed investigations are needed to map quantitative differences in the transcription and to further assess the complex regulatory network of transcription in *E. chaffeensis*.

The current study provides the first evidence suggesting that *E. chaffeensis *whole-cell protein lysates contain regulatory proteins which modulate transcription of *p28-Omp14 *and *p28-Omp19 *promoters *in vitro*. In support of further testing the hypothesis that *E. chaffeensis *whole-cell protein lysates contain proteins that bind to putative regulatory DNA sequences of these promoters, EMSA experiments were performed. A shift in mobility of DNA fragments was observed for several partial or complete DNA segments of the promoter regions of both *p28-Omp14 *and *p28-Omp19 *genes. These data suggest that the promoter region contained regulatory DNA sequences that allowed binding of one or more *E. chaffeensis *proteins. The binding was specific as the addition of specific competitors considerably reduced the shift and the addition of a non-specific protein did not cause a shift. The binding of *E. chaffeensis *regulatory proteins to the DNA segments spanning putative DNA binding elements is consistent with previous studies on this organism [49] as well as in several other bacteria, including *Anaplasma phagocytophilum *[50-52], *C. trachomatis *[34,35]and *B. subtilis *[53,54]in which interaction of regulatory proteins with regulatory sequences have been demonstrated. The identity of DNA binding proteins and the location of protein binding sites remain to be determined.

## Conclusions

In this study, we developed *in vitro *transcription assays using a G-less cassette and described methods to isolate native RNAP and the recombinant RNAP σ70 subunit of *E. chaffeensis*. The value of using these tools in evaluating the promoters of two differentially expressed genes has been demonstrated. The application of these tools to the study of *E. chaffeensis *is new and important for furthering our understanding of the regulation of gene expression in this pathogen. Specifically, the tools will be valuable in studies to map specific interactions of *E. chaffeensis *proteins in driving differential gene expression influenced by vertebrate and tick host cell environments. This is the first report of *in vitro *transcription using native *E. chaffeensis *RNAP and *E. coli *RNAP core enzyme reconstituted with the recombinant *E. chaffeensis *σ^70 ^subunit. This study marks the beginning of a greater effort to broadly characterize the mechanisms that control the transcription in *Anaplasmataceae *pathogens in support of their growth in vertebrate and tick hosts.

## Methods

### PCR conditions

PCRs for amplification of *E. chaffeensis p28-Omp14 *and *p28-Omp19 *promoters were carried out in a 25 μl reaction volume containing 0.2 μM of each primer, 250 ng of purified *E. chaffeensis *(Arkansas isolate) genomic DNA, 400 μM of each of the four deoxyribonucleoside triphosphates, 1.5 mM MgSO_4_, 1x native HiFi PCR buffer (60 mM Tris-SO_4_, 18 mM (NH_4_)_2_SO_4_), 2.5 units HiFi polymerase. After the first denaturation step of DNA at 95°C for 2 min, amplification was carried out for 45 cycles of denaturation at 95°C for 30 s, annealing at 40°C for 30 s and extension at 72°C for 50 s and a final extension at 72°C for 2 min.

### Construction of transcription plasmids

The plasmid pMT504 is a G-less cassette plasmid containing two transcription templates cloned in opposite directions to aid in driving transcription from promoters introduced upstream of the G-less cassette sequences [26]. We constructed *in vitro *transcription templates, pRG147 and pRG198, by cloning the promoter regions of *p28-Omp14 *and *p28-Omp19*, respectively, into the pMT504 plasmid at EcoRV site (Figure 1). The promoter sequences selected for preparing these constructs included the sequences starting from the downstream first nucleotide of the termination codon of the upstream gene and up to the transcription start sites of the genes mapped in our previous study [25]. Plasmid pRG147 contained a 553 bp promoter region of *p28-Omp14 *amplified from genomic DNA using primers RRG217 and RRG695 (Table 1). Similarly, plasmid pRG198 contained a 306 bp promoter region of *p28-Omp19 *amplified by primers RRG185 and RRG696. All oligonucleotide primers used in this study were designed from the genome sequence data [24] and were synthesized at Integrated DNA Technologies, Inc. (Coralville, Iowa). Reverse primers for promoter segments included the transcription start sites of the respective promoters but excluding any guanosine residue downstream of the transcription initiation sites. This is to avoid transcription termination caused by incorporation methylated guanosine triphosphate present in the transcription reactions (outlined below under *in vitro *transcription). The promoter inserts were also cloned in opposite orientation (pRG147R and pRG198R) to serve as negative controls to demonstrate promoter-specific *in vitro *transcription.

Transcription from pRG147, pRG198 or pMT504 plasmids results in a shorter 125-nucleotide transcripts encoded by a control transcription template positioned downstream of the *Chlamydia trachomatis *rRNA P1 promoter. The test transcription template contains a 153-nucleotide G-less cassette segments in the opposite direction to the control transcription template. This synthetic template results in the transcription of a 162-nucleotide transcript from the transcription start site for both the p28-Omp14 and 19 gene promoters. Supercoiled plasmids for use in the *in vitro *transcription assays were prepared using the QIAprep Spin Miniprep kit (Qiagen Inc., Valencia, CA) according to the manufacturer's instructions. The DNA sequences of the promoter templates were verified by restriction enzyme and sequencing analysis.

### *In vitro *transcription assays

*In vitro *transcription reactions were performed in a 10 μl final reaction volume with the following components; 50 mM Tris-acetate buffer pH 8.0 containing 50 mM potassium acetate, 8.1 mM magnesium acetate, 27 mM ammonium acetate, 80 mM NaCl, 2 mM DTT, 400 μM ATP, 400 μM UTP, 2.1 μM [α-^32^P]-CTP (800 Ci mmol^-1 ^for radioisotope detection method) or 400 μM CTP (for detection and quantification by real-time reverse transcription PCR), 100 μM sodium salt of 3'-O-methylguanosine 5'-triphosphate, 18 units of RNasin, 5% glycerol, 0.13 pmol of supercoiled DNA template and 1 μl (360 ng) of heparin-agarose purified *E. chaffeensis *RNAP or 0.5 μl of 1:10 dilution of *E. coli *core enzyme (Epicenter, Madison, WI) or 0.5 μl of 1:10 dilution of *E. coli *σ^70^-saturated holoenzyme (Epicenter, Madison, WI). For enzyme salt tolerance assays, potassium acetate and NaCl concentrations were varied over a range from 0 to 600 mM and 0 to 120 mM, respectively. In transcription reactions using *E. chaffeensis *recombinant σ^70^, RNAP holoenzyme was reconstituted by adding 360 ng of recombinant protein to 0.5 μl of 1:10 diluted *E. coli *core enzyme. Holoenzyme formation was allowed to occur by incubating the mixture on ice for 20 min. To assess the modulatory effect on transcription, 4.0 μg of *E. chaffeensis *protein lysate (preparation described below) was incubated for 20 min at room temperature with the transcription reaction mixture in the absence of an RNAP to allow binding of proteins to DNA elements of promoter segments. Next, 1 μl of the purified *E. chaffeensis *RNAP was added to reaction mixture. In general, transcription reactions were incubated at 37°C for varying times of 7.5 min, 15 min or 30 min and the reactions were terminated by adding 7 μl of stop solution (95% formamide, 20 mM EDTA, 0.05% bromophenol blue and 0.05% xylene cyanol). Six microliters of the sample was electrophoresed on a 6% polyacrylamide sequencing gel containing 7 M urea. The gels were dried and transcripts were visualized by exposing an X-ray film to the gels. Autoradiographs were scanned on a HP SCANJET 5550 scanner (Hewlett-Packard^®^).

### Isolation of *E. chaffeensis *RNAP

The RNAP isolation method was a modified version from the heparin-agarose procedure described in [21,27,55]. *E. chaffeensis *Arkansas isolate was grown in confluent DH82 cells (malignant canine monocyte/macrophage cells) in 300 cm^2 ^culture flasks in 1 litre MEM tissue culture medium containing 7% fetal bovine serum (Gibco BRL^®^) and 1.2 mM L-glutamine [56]. DH82 cultures infected with *E. chaffeensis *having predominantly reticulate bodies (RB) were harvested 48 h post-infection by centrifugation at 1,000 × g for 10 min at 4°C in an Eppendorf 5810R centrifuge. (All centrifugation steps were performed using this centrifuge.) The purification steps were all performed at 4°C. The pellet was resuspended in 25 ml sucrose potassium glutamate (SPG) buffer (218 mM sucrose, 3.76 mM KH_2_PO_4_, 7.1 mM K_2_HPO_4_, 5 mM potassium glutamate, pH 7.0) and host cells were lysed in a 40 ml Wheaton homogenizer with pestle A. The lysate was centrifuged at 800 × g for 10 min in 50 ml conical tubes to pellet host cell debris. Subsequent supernatant was centrifuged at 15,000 × g for 10 min to pellet the organisms. The RB pellet was resuspended in 2 ml of freshly prepared lysis buffer [10 mM Tris-HCl (pH 8.0), 10 mM MgCl_2_,1 mM EDTA, 0.3 mM dithiothreitol (DTT), 7.5% glycerol (vol/vol), 50 mM NaCl, 1x Amersham protease inhibitor mixture, and 150 μg per ml of lysozyme]. Lysis was facilitated by three passages through 27.5 G needle. Sodium deoxycholate (at final concentration of 0.05%) was added to the lysate and the suspension incubated for 30 min at 4°C. The lysate was centrifuged at 10,000 × g for 10 min and the supernatant was collected and clarified by an additional centrifugation step for 5 min.

The clarified supernatant was loaded onto pre-packed heparin-agarose column (type I-S, Sigma^®^) previously equilibrated with buffer A [10 mM Tris HCl (pH 8.0),10 mM MgCl_2_,1 mM EDTA, 0.3 mM DTT, 7.5% glycerol and 50 mM NaCl]. The suspension was adsorbed for 60 min at 4°C and the column was washed by gravity with 20 ml of buffer A for complete removal of unbound proteins. The bound proteins from the column were eluted by gravity with buffer A containing 0.6 M NaCl and 0.5 ml fractions were collected. Based on previous analysis and calculation of the void volume of the column, fractions 3-6 were pooled and dialyzed overnight against storage buffer [10 mM Tris-HCl (pH 8.0), 10 mM MgCl_2_, 0.1 mM EDTA, 0.1 mM DTT, 50% glycerol and 100 mM NaCl] using Slide-A-Lyzer Gamma Irradiated Dialysis Cassette (Thermo Scientific, Illinois, USA). The fractions were stored at -80°C. RNAP activity of the dialyzed fraction was determined by *in vitro *transcription assay.

### Protein concentration

Protein concentration of the HA purified RNAP fractions and *E. chaffeensis *whole-protein lysates were measured with the bicinchoninic acid protein assay reagent (Thermo Scientific, Illinois, USA) with bovine serum albumin as the protein standard.

### SDS-PAGE

Proteins were analyzed by electrophoresis in 7.5% sodium dodecyl sulphate-polyacrylamide gel (SDS-PAGE), followed by silver staining according to the procedures provided by the manufacturer (G Biosciences, USA) or resolved proteins were transferred onto a nitrocellulose membrane, Hybond-ECL (Amersham Biosciences, Germany), for immunoblot analysis.

### Western blot (immunoblot) of RNAP extracts

*E. chaffeensis *RNAP purified above was subjected to SDS-PAGE and the proteins were electroblotted for 2 h at 70 V to a sheet of nitrocellulose membrane. The membrane blot was blocked in a solution containing 10% nonfat dried milk (NFDM) freshly made in TTBS [0.1% Tween-20 in 100 mM Tris-HCl (pH 7.5) and 0.9% NaCl] for 1 h at room temperature with gentle agitation. The blot was rinsed three times in TTBS and then was incubated for 1 h at room temperature or overnight at 4°C with anti-*E. coli *σ^70 ^antibody, 2G10 (Santa Cruz Biotechnology Inc., Santa Cruz, CA), diluted 1: 500 in 1% NFDM in TTBS solution. The blot was washed again three times with washing solution and then incubated for 1 h at room temperature with horseradish peroxidase-conjugated anti-mouse immunoglobulin G diluted 1:5000 in 1% NFDM in TTBS solution. The blot was rinsed again three more times with TTBS to remove excess secondary antibody and detection was carried out using chemiluminescent detection reagents (Amersham ECL™, GE Healthcare).

### Properties of isolated *E. chaffeensis *RNAP

Assays to determine the salt tolerance of the purified enzyme have been described above. Rifampin/rifampicin is a potent inhibitor of prokaryotic RNAPs, but not for eukaryotic RNAP [27]. As *E. chaffeensis *RNAP was recovered from organisms grown in eukaryotic cells (DH82), it may be potentially contaminated with eukaryotic RNAP. To confirm that the transcript formation is from *E. chaffeensis *RNAP but not from eukaryotic RNAP, *in vitro *transcription assays were performed in the presence of rifampin at a concentration of 25 μg ml^-1^.

Functional studies with an *E. coli *RNAP monoclonal antibody (2G10) demonstrated that it can effectively bind to *E. coli *σ^70 ^and markedly inhibit *in vitro *transcriptional activity by RNAPs of *E. coli *[29] and *C. trachomatis *[28]. To further assess that *in vitro *transcriptional activity was due to *E. chaffeensis *purified RNAP but not from eukaryotic RNAP, we utilized the *E. coli *monoclonal antibody 2G10 in inhibition assays assuming that it blocks the *E. chaffeensis *RNAP similar to *C. trachomatis *RNAP. For this experiment, 4 μg of 2G10-antibody was added in transcription reactions and the production of transcripts were assessed by following the methods described above.

### Overexpression and purification of *E. chaffeensis *RpoD (σ^70^)

The entire RpoD (σ70 subunit gene) protein coding sequence, identified from the *E. chaffeensis *Arkansas isolate genome [24], was amplified by PCR and cloned into the pET32 plasmid (Novagen, Madison, WI) for producing recombinant protein. The PCR was performed using *pfu *DNA polymerase (Promega, Madison, WI) and with the gene-specific PCR primers, RRG742 and RRG 743 (Table 1). To facilitate directional cloning, *Nco*I and *Xho*I restriction enzyme sites were engineered in the PCR product. The PCR product was subsequently cloned into pET32 plasmid at the above restriction sites after digesting both plasmid and inserts and ligating using T4 DNA ligase. Over expression of RpoD protein and its purification was carried out with methods similarly described elsewhere [20,57]. The concentration of the purified RpoD protein was approximately 180 ng/μl, as determined by protein estimation method (described above).

### Quantification of transcription

We carried out quantification of *in vitro*-generated RNA transcripts of *p28-Omp14 *and *p28-Omp19 *promoters by densitometry and TaqMan probe-based real-time RT-PCR. For densitometric analysis, we quantitated the signal intensity of radio actively labelled transcripts on X-ray films using ImageQuant software 5.2 (Molecular Dynamics, Inc., Sunnyvale, CA). For real-time RT-PCR analysis, primers and TaqMan probes for the 162 and 125 nucleotide (nt) G-less cassettes were designed manually and optimized using Vector NTI Advance 11 software (Invitrogen, Carlsbad, CA). The primers and probes used for these assays were listed in Table 1. The TaqMan probe for the 162 nt cassette (RRG765) and the probe for the 125 nt cassette (RRG768) have been labelled with reporter fluorescent dyes TET and ROX and quencher dyes Iowa Black FQ and Iowa Black RQ-Sp, respectively. Real-time RT-PCR was carried out using the SuperScript™ III One-Step RT-PCR reagents (Invitrogen, Carlsbad, CA). Each RT-PCR reaction contained the following: 1x reaction mix (containing 200 μM dNTPs), 5 mM MgSO_4_, 100 nM of each primer, 150 nM of each TaqMan probe, 1 μl of SuperScript III reverse transcriptase/Platinum *Taq *mix and 1 μl of *in-vitro *transcribed RNA sample in a 25 μl volume. Reverse transcription was carried out for 30 min at 48°C followed by a denaturation step of 2 min at 95°C. The PCR amplification was then performed for 40 cycles with each cycle at 94°C for 15 s and 60°C for 30 s. All reactions were carried out in triplicate using a Smart Cycler system (Cepheid, Sunnyvale, CA). The threshold cycle, C_t_, values of the samples (containing 4.0 μg of *E. chaffeensis *protein lysate) were averaged from values obtained from each reaction, and the promoter activity was calculated as a relative level of expression to the reference control in a separate tube. The relative level of expression was calculated using the mathematical model of relative expression ratio in real-time PCR under constant reference gene expression [31]: Ratio = (*E*_target_)^ΔCT^_target__*(control-sample)*_, where *E *represents the PCR efficiency of one cycle in the exponential phase and was calculated according to the equation: *E *= 10^[-1/slope]^.

### Preparation of *E. chaffeensis *whole-cell soluble protein lysates

*E. chaffeensis *organisms were cultivated *in vitro *in canine macrophage (DH82) cell lines at 37°C or in ISE6 tick cells as described previously [18,56]. The protocols for *E. chaffeensis *cell lysate preparations were similar to previously described methods for *E. chaffeensis, A. phagocytophilum *and other Gram negative bacterial organisms [49,52,58]. Twenty five ml of about 80-100% *E. chaffeensis *infected cultures were harvested using glass beads. The cultures were centrifuged at 15,560 × *g *for 15 min to recover infected host cells and cell free *E. chaffeensis *organisms. To release the organisms from host cells, the pellet was resuspended in 10 ml SPK buffer (0.5 K_2_HPO4, 0.5 M KH_2_PO4, and 0.38 M sucrose) and sonicated twice for 30 sec at a setting of 6.5 in a Sonic Dismembrator (Fisher Scientific, Pittsburgh, PA). The cell lysates were centrifuged at 400 × *g *for 5 min and the supernatant containing cell free *E. chaffeensis *was filtered through a 5 μm and 3 μm sterile isopore membrane filters (Millipore, Billerica, MA). The filtrate containing cell free organisms was centrifuged at 15,560 × *g *for 15 min at 4°C. The pellet containing *E. chaffeensis *organisms was washed twice with 1.5 ml of lysis buffer (150 mM Tris-HCl pH 8.0, 100 mM KCl, 10 mM Magnesium Acetate, 1 mM EDTA, 2 mM DTT and 10% glycerol) and the pellet was resuspended in 1 ml of lysis buffer containing protease inhibitors (Roche Diagnostic Labs, Indianapolis, IN). The cell suspension was sonicated four times at 8.5 setting, 30 sec each time to lyse *E. chaffeensis *organisms. The cell lysates were centrifuged at 15,560 × *g *for 15 min at 4°C to pellet the insoluble fraction and the supernatant containing soluble proteins of *E. chaffeensis *was collected into sterile micro centrifuge tubes as 25 μl aliquots containing protease inhibitor mix and stored at -80°C until use. Protein concentration of the protein lysates, prior to adding the protease inhibitor mix, was estimated as described above.

### Electrophoretic mobility shift assay (EMSA)

DNA sequence segments spanning one or more putative regulatory sequences of *p28-Omp14 *or *p28-Omp19 *gene promoters were amplified from *E. chaffeensis *Arkansas isolate genomic DNA using sequence specific primers and 5'end biotin-labeled reverse primers (Table 1) and evaluated for their interaction with the protein lysates. EMSA experiments and detection were carried out according to established protocols [57,58] with a radioactive nucleotide incorporated DNA probes or using the LightShift Chemiluminescent EMSA kit (Pierce Biotechnology, Rockford, Illinois, USA) according to the specifications of the manufacturer. The assay mixtures included a non-specific DNA (salmon sperm DNA or poly dI.dC at a high concentration of 240 μg/ml or 50 μg/ml, respectively) to eliminate non-specific interactions. Briefly, about 1 ng of each of the full length or biotin-labeled partial upstream sequences was used in each reaction together with 5 μg of the *E. chaffeensis *whole-cell protein lysate. About 50 ng of unlabeled specific probe sequences were used as competitors. Bovine serum albumin (BSA) was included in each experiment as a non-specific protein control. The protein concentration in *E. chaffeensis *protein lysates used in these experiments was similar to the work reported earlier [41,49,58].

### Statistical analysis

We carried out two-tailed t-tests with equal variances for densitometry analysis and unequal variances for the real-time RT-PCR analysis to comparatively analyse the effect of addition of *E. chaffeensis *whole cell protein lysate on transcription of *p28-Omp14 *(pRG147) and *p28-Omp19 *(pRG198) promoters.

## Authors' contributions

BF carried out the native RNAP isolation, bioinformatics analysis, *in vitro *promoter mapping studies, statistical analysis, and drafted the manuscript and compiling the appropriate references. HL prepared the recombinant σ70 subunit and participated in the *in vitro *promoter mapping studies using *E. coli *RNAP reconstituted with the recombinant protein. LP carried out EMSA experiments. RRG conceived of the study and participated in its design and coordination, instrumental in obtaining financial support, helped in data analysis and to draft the manuscript to its final form. All authors read and approved the final manuscript.
